# Reliability and Validity of the 20-Item Toronto Alexithymia Scale in Korean Adolescents

**DOI:** 10.4306/pi.2009.6.3.173

**Published:** 2009-07-15

**Authors:** Sang Soo Seo, Un-Sun Chung, Hyo Deog Rim, Sung Hoon Jeong

**Affiliations:** 1 Department of Psychiatry, School of Medicine, Kyungpook National University, Daegu, Korea.; 2 Department of Psychiatry, Kyungpook National University Hospital, Daegu, Korea.

**Keywords:** Toronto alexithymia scale, Alexithymia, Adolescents

## Abstract

**Objective:**

Adolescence is a period of developing emotional regulation. However, alexithymia has rarely been examined during this period. The objective of this study is to examine the factor structure and internal consistency of the Korean version of the 20-item Toronto Alexithymia Scale (TAS-20K) in normal adolescents in South Korea.

**Methods:**

The TAS-20K was administered to a sample of 290 adolescents aged from 12 to 16 years old. Internal reliability, test-retest reliability, and factorial validity were evaluated.

**Results:**

The three factors of the TAS-20K were confirmed by confirmatory factor analysis. The internal consistency, measured using Cronbach's alpha coefficient was acceptable for difficulty in identifying feelings, good for difficulty in describing feelings, and acceptable for externally oriented thinking.

**Conclusion:**

Our study indicates that the TAS-20K is an appropriate instrument to assess alexithymia in Korean adolescents.

## Introduction

The term "alexithymia", which literally meant "no words for feelings," was originally introduced by Sifneos in the 1970s, when he described the emotional deficits among psychosomatic patients.^1^ Over the last 3 decades, the alexithymia construct was refined theoretically, with an accumulating body of research supporting the view that the features of the construct reflected a deficit in cognitive processing and emotional regulation.^2^,^3^ The salient features were 1) difficulty in identifying and describing feelings, 2) difficulty in distinguishing between feelings and the bodily sensations of emotional arousal, 3) constricted imaginal processes, and 4) externally oriented cognitive style.^2^,^4^ Much evidence suggested that alexithymia was strongly associated with several common medical and psychiatric disorders, such as functional gastrointestinal disorders, substance use disorders, post-traumatic stress disorders, eating disorders, and medically unexplained physical symptoms.^2^,^5^-^8^

Many researchers have attempted to develop reliable and valid instruments to measure alexithymia, using varied approaches including self-report scales, observer-rated questionnaires, and projective techniques. Most of these early instruments, however, failed to meet minimal psychometric standards.^2^,^4^ At the present time, measures with adequate psychometric properties include the Bermond-Vorst Alexithymia Questionnaire,^9^ the Observer-Alexithymia Scale,^10^ the Toronto Structured Interview for Alexithymia,^11^ a set of Rorschach variables,^12^ and the 20-item Toronto Alexithymia Scale (TAS-20).^13^ From this methodologically varied list, by far the one most widely used in both research and clinical practice is the self-report TAS-20.^13^-^15^ The TAS-20 has a three-factor structure that has been replicated in student, community, and clinical samples, and has also been cross-validated in many different languages and cultures^13^-^17^ including in Korean.

The cognitive level of children does not allow them to have the abstraction and self-reflection that is needed to recognize and describe their emotions and associations connected with stressors.^18^ This is why children with emotional distress often show psychosomatic symptoms. The cognitive capacities for abstraction and reflection on emotion and the sensations of the body rapidly increase toward an adult type during adolesence.^19^ From childhood to adolescence, the ability to recognize and understand emotions and to verbalize emotions also increases.^20^ This suggests that alexithymic tendencies would decrease as adolescent development advances. If such normal cognitive and emotional development, however, does not occur, such adolescents would express their emotions in a relatively immature way for their age, and it is important for clinicians to examine alexithymic tendencies when they treat adolescents with psychiatric disorders. Horton et al.^21^ compared a group of severely alexithymic adolescents with normal adolescents and found the former to have significantly less ability to use psychological methods of self-comforting such as memories or interaction with other people than normal, non-alexithymic subjects of the same age. Rieffe et al.^22^ also supported the idea that core features of alexithymia can be identified and measured both in childhood and adolescence. They found certain children aged 11 to 13 reported somatic complaints and negative moods frequently associated with alexithymia. Until now, little research for measuring alexithymia in adolescents has been carried out. As far as we know, there is only one study evaluating the psychometric properties of the TAS-20 in a sample of 12- to 17-year-old adolescents in Finland.^23^ The aim of this study is to evaluate the reliability and validity of the Korean version of the 20-item Toronto Alexithymia Scale (TAS-20K) in Korean adolescents aged from 12 to 16 years old.

## Methods

### Participants

The questionnaire was administered to 310 middle school students. They were a non-clinical sample and 290 (93.5%) of the participants provided complete data. Thus, this study included 290 adolescents (147 boys, 50.7%; 143 girls, 49.3%) ranging in age from 12 to 16 years old (mean, 13.46; SD, 0.89) in Daegu City, which has approximately 2.4 million inhabitants, located in southeastern South Korea. We sent letters to headmasters requesting their school's participation in this study assessing the reliability and validity of the TAS-20K in Korean adolescents. Permission was obtained in advance from the headmasters, teachers, and parents' committees of the school districts in which the study was performed. The study sample was drawn from two middle schools representing different socioeconomic areas of the city. Participants with prior psychiatric diagnosis and treatment have been excluded. The sample size was estimated according to the following general rule: to have at minimum five times as many observations as variables to be analyzed, and preferably a ten-to-one ratio.^24^

To estimate test-retest reliability, 22 randomly selected students were asked to complete the same scale after a 4-week interval. All participants were volunteers who were informed that the aim of the research was to evaluate the reliability and validity of the TAS-20K. Written informed consent was provided by all participants at enrollment.

### Instrument

The TAS-20 is a 20-item self-report instrument with each item rated on a 5-point Likert scale ranging from 1 (strongly disagree) to 5 (strongly agree); 5 items are inversely rated. Total scores range between 20 and 100, and higher scores mean a higher tendency toward alexithymia. For the English version of the scale, cutoff scores have been established empirically with total scores greater than 60 indicating a presence of alexithymia and scores less than 52 indicating a definite absence of alexithymia.^25^ The TAS-20 consists of three factors: 1) 7 items for difficulty in identifying feelings and distinguishing them from the bodily sensations of emotions (DIF); 2) 5 items for difficulty in describing feelings to others (DDF); 3) 8 items for an externally oriented cognitive style of thinking (EOT).

The reliability and validity of TAS-20K have been well-demonstrated in adults by Lee et al.^26^ in 1996 and Chung et al.^27^ in 2003. The first study showed that one item (#5: "I prefer to analyze problems rather than just describe them") had very low factor loading on factor 3 due to cultural differences. Lee et al.^26^ observed that the expression, 'analyze' contains a purely logical meaning without emotional understanding in the Korean language, and suggested that a revision of that item ("I prefer to try to understand problems rather than just describe them") would be appropriate. The original author of the Korean scale agreed with that opinion and named the Korean version of the TAS-20, the TAS-20K. Chung et al.^27^ proved that, in Korean culture, the revised version of the TAS-20K was more valid than the first one.

### Statistical procedures

To determine how well the original three-factor TAS-20 model fits the Korean data in the adolescent sample, we performed a confirmatory factor analysis (CFA) using LISREL version 8.1.28 To examine the model fit, we evaluated 4 fit indices: the Goodness-of-Fit Index (GFI); the adjusted goodness-of-fit index (AGFI); the root-mean-square residual (RMS); and the Root-Mean-Square Error of Approximation (RMSEA). The following standards were used to evaluate the model fit: GFI 0.85 or greater; AGFI 0.80 or greater; RMS 0.10 or less; and RMSEA 0.08 or less.^29^-^33^ Confirmatory factor analysis (maximum likelihood estimation, with an oblique method) was done for the TAS-20K in a Korean adolescent sample.

To evaluate internal reliability and item-to-scale homogeneity of the TAS-20K in a Korean adolescent sample, we calculated Cronbach's α coefficients and mean inter-item correlations for the total scale and for each factor. A standard of 0.70 or higher was set for α, and an optimal range of 0.20 to 0.40 for the mean interitem r.^34^,^35^ Test-retest reliability was evaluated using Pearson correlation r, with a minimal standard of 0.70.^36^ The relations between the total score and the three factors were also examined using Pearson correlation r.

The comparisons of the TAS total score and subscale scores between boys and girls were estimated by use of an independent t-test and among the three age-groups by one-way analysis of variance (ANOVA). In order to compare alexithymia across age groups, the sample was divided into three groups: group 1 (12-13 years old), group 2 (14 years old), and group 3 (15-16 years old). The basis for choosing these categories was to create groups of similar sizes, in order to ensure sufficient data for all analyses, stratified by age as in the Finnish adolescent study.^23^ Of the subjects, 33.8% were in the youngest age-group, 35.9% in the second, and 30.3% in the oldest group.

The Bonferroni test was used as a post-hoc test for the ANOVA. The Pearson χ^2^ test was used to test differences in the rate of alexithymia between groups, and the Pearson χ^2^ test with Bonferroni correction was used as a post-hoc test. Statistical analysis was performed with SPSS/Windows (Version 14.0).

## Results

### Construct validity and confirmatory factor analysis

The fit indices of the standard three-factor model reached acceptable standards, GFI=0.898, AGFI=0.863, RMS=0.072, and RMSEA=0.061 (90% confidence interval, 0.052-0.071). Parameter estimates for the whole sample are presented in Table 1.

We used the common three-factor solution, which is presented in Table 2. The GFI, AGFI, RMS and RMSEA showed a good fit with the data for all the different subgroup models. The parameter estimates for the relationships among the three factors are also presented in Table 2. For the whole sample, the estimate between Factors 1 (DIF) and 2 (DDF) was 0.91, between Factors 1 (DIF) and 3 (EOT), 0.44, and between Factors 2 (DDF) and 3 (EOT), 0.59. Although the estimate between Factors 1 and 3 of 0.27 and the estimate between Factor 2 and 3 of 0.33 for girls were low, all estimates for various subgroups were significant (p<0.05).

### Reliability

As shown in Table 3, the Cronbach's α coefficient for the full TAS-20K was 0.87 for the non-clinical adolescent sample. The Cronbach's α coefficient for the three factors ranged from 0.61 (DDF) to 0.85 (DIF). The mean inter-item correlation coefficient was 0.26 for the 20 items of the TAS-20K. The mean inter-item correlation coefficient for the three factors ranged from 0.25 (DDF) to 0.40 (DIF). The test-retest coefficient for the TAS-20K was 0.87. For the three factors, the test-retest coefficients were 0.89 (DIF), 0.65 (DDF), and 0.72 (EOT), respectively.

### Alexithymia in adolescents

The mean total score on the TAS-20K was 45.7 (SD: 8.5). The mean score for boys was 46.2 (SD: 8.7) and for girls was 45.1 (SD: 8.2) with no difference between the sexes. There were no significant differences in the mean scores among age-groups.

The mean scores of the three TAS factors were the following: DIF: 11.9 (SD: 4.9); DDF: 11.5 (SD: 3.3); and EOT: 22.3 (SD: 3.6). The differences were not statistically significant between the sexes for the three TAS factors. The differences were statistically significant between age-groups for DIF and EOT factors.

Among 290 middle school students, 5.2% (5.4% of boys and 4.9% of girls; p=0.83) were alexithymic. In the youngest age-group, the rate of alexithymia was 4.1% (boys: 4.4%, and girls: 3.8%), and in the middle and oldest groups, 6.7% (boys: 7.8%, and girls: 5.7%) and 4.5% (boys: 3.9%, and girls: 5.4%), respectively. The difference between the age-groups for the rate of alexithymia was not significant (p=0.66). No significant differences emerged between the sexes in any age-group for the rate of alexithymia. Descriptive statistics of total scores and the three factors of the TAS-20K are presented in Table 4. Alexithymia rates in adolescents were calculated using cutoff scores for adults, because no study has yet been done to produce the cutoff scores for adolescents.

## Discussion

In general, the results of this study support the idea that the TAS-20K has good psychometric properties in adolescent subjects. The estimates of internal reliability for the full scale are comparable to those obtained with the English version of the scale (TAS-20: 0.86, DIF: 0,80, DDF: 0.76, EOT: 0.71).^13^,^15^,^16^ They have better internal reliability than the results obtained with a university student sample in Korea, (TAS-20K: 0.76, DIF: 0,79, DDF: 0.65, EOT: 0.49)^26^ and the results of a Finnish adolescent sample (TAS-20: 0.73, DIF: 0,78, DDF: 0.64, EOT: 0.57).^23^

According to the original authors of the English scale, factor 3 is consistently reported to have low internal consistency, and Cronbach's α coefficients for factor 3 mostly ranged between 0.34 to 0.68 for different language translations.^16^ Taylor et al.^16^ explained that factor 3 has the greatest number of items which influence internal reliability and has more negatively keyed items, and that altering meaning in the translation process or true cultural differences can contribute to this finding. In the TAS-20K, after revision of item 5 regarding Korean culture, the factor weight of item 5 on factor 3 (EOT) had been improved from 0.11 to 0.40, and the Cronbach's α coefficient for factor 3 had been improved from 0.49 to 0.59 in the university student sample.^27^ In our study, the factor weight of item 5 of 0.64 was higher than in the Korean university student sample (0.40) and the Cronbach's α coefficient, 0.74 for factor 3, was also higher than that of the Finnish adolescent sample (0.57).^23^ These results suggest that revision in light of cultural differences between Western and Korean culture is more appropriate for evaluation of Korean adolescents, and it provides more reliable and valid results for factor 3 and the total TAS-20K administered to Korean adolescents.

Although the internal reliability estimates (α=0.61) are below the standard of 0.70 for the DDF factors of the present study, the original author of the English scale sugested that the Cronbach's α coefficient above 0.60 deonstrated adequate reliability for factor 2 (DDF) and 3 (EOT).^16^ Moreover, the mean interitem correlation for DDF (0.25), which assessed the homogeneity of a scale and its factor, is within the optimal range of 0.20 to 0.40.^16^ This means that the DDF factor is unidimensional.^37^

The parameter estimates of the factor relationship for girls were higher than for boys, and the estimates related to Factor 3 for girls were low. These results are consistent with the findings of a Finnish adolescent sample and a university student sample in Korea.^23^,^26^,^27^

The originally established three-factor model for the TAS-20^13^,^14^ was replicated in our study and four criteria of goodness-of-fit reached the standards for adequacy of fit.

Although the test-retest reliability was less than 0.70 for the DDF factor (0.65), TAS-20K total scores and DIF and DDF scores of the TAS-20K were stable over a 4-week period.

Adolescence is the period of maturation when emotional, psychological, and social development proceeds. A capacity to be aware of one's own emotions and feelings and to regulate them increases more rapidly during adolescence than in childhood. We hypothesized that the incidence of alexithymia would decrease apace in the process of adolescent development, compatible with the results of a previous study.^23^ A decrease in mean scores, however, was only seen from 12- to 16-year olds for EOT and an increase in mean scores was seen from 12- to 16-year olds for TAS total scores and DIF and DDF scores. In fact, the Korean samples showed a lower alexithymia rate than the Finnish samples.^23^ The Korean adolescent sample (290 adolescents aged from 12 to 16 years) was 5.2% and the Finnish adolescent sample (882 adolescents aged from 12 to 17 years) was 15.9%. The reason for this might be attributable to cultural differences. Given that culture can have a profound influence on the experience and expression of emotions, it follows that culture might influence components of the alexithymia construct, in particular the identification and communication of subjective feelings.^38^

This study showed no significant differences among the age-groups in the prevalence of alexithymia. This result is inconsistent with the findings of a previous study.^23^ This finding may be accounted for by the increasing mean total TAS-20K scores, DIF scores and DDF scores with age. Recently, a study on the prevalence of depression in Korean adolescents was conducted, and it showed that depressive symptom rates increase with grade level in school.^39^ Because alexithymic tendencies are associated with depressed mood, the results of the present study might be unrevealed normal cognitive and emotional regulative development.

More cross-cultural studies are needed to evaluate possible changes in alexithymic tendencies over developmental periods. Knowledge of normative alexithymic featu res can be helpful in assessing a given adolescent's development, and especially in assessing an adolescent's age-appropriate capacity to do "psychotherapeutic work" and in providing appropriate treatment including psychotherapy. Because lack of emotional regulation capacity is associated with psychiatric problems,^2^,^5^-^8^ such knowledge also can be helpful in assessing an adolescent's psychiatric disorders, planning for treatment, and tracing prognosis. Moreover, this scale can be helpful in assessing emotional awareness and regulation of adolescent sexual offenders, adolescents with anorexia nervosa, and even adolescents with Asperger's syndrome in Korea.^40^-^42^

This study has limitations: it was not intended to measure sociodemographic or depression factors. Both of them have been shown to be associated with alexithymia among adults.^43^,^44^ This study included only self-reported data. In the future, these results should be confirmed in further studies by using alternative methods for assessing alexithymia, such as the recently developed Toronto Structured Interview for Alexithymia,^11^ which provides a deeper knowledge of alexithymia in adolescence.

TAS-20 total sores >60 are defined as alexithymic cases. The study used this common cutoff point to calculate the alexithymic rate for adolescents. However, this point has not been validated for adolescents yet. Further studies are needed to label a cufoff point for adolescent-aged subjects.

The results of this study support the use of the TAS-20K for a Korean sample of adolescents. Future research should evaluate the concurrent, convergent, and discriminant validity of alexithymia measures, and to explore the extent to which alexithymia might influence health among Korean adolescents.

We conclude that the TAS-20K is an appropriate method for assessing alexithymia among young people in Korea.

## Figures and Tables

**TABLE 1:**
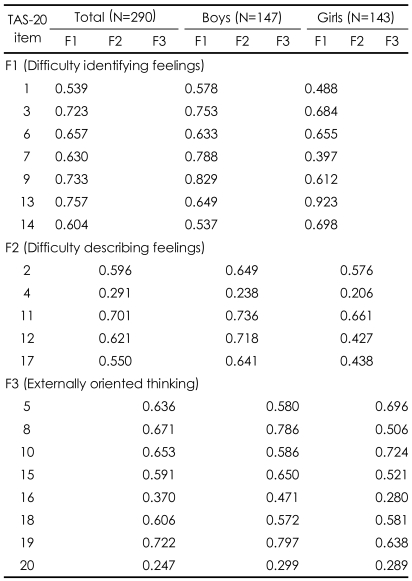
Parameter estimates from the results of confirmatory factor analyses of the TAS-20K among non-clinical adolescents aged from 12 to 16 years

TAS-20K: the Korean version of the 20-item Toronto Alexithymia Scale, N: number

**TABLE 2:**
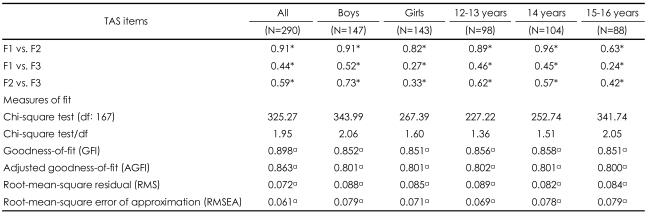
Confirmatory factor analysis: parameter estimates for the relationships among the three factors of the TAS-20K and goodness-of-fit indices among non-clinical adolescents aged from 12 to 16 years old

^*^p<0.05. F1: difficulty in identifying feelings, F2: difficulty in describing feelings, F3: externally oriented thinking a accepted value. N: number TAS-20K: the Korean version of the 20-Item Toronto Alexithymia Scale

**TABLE 3:**
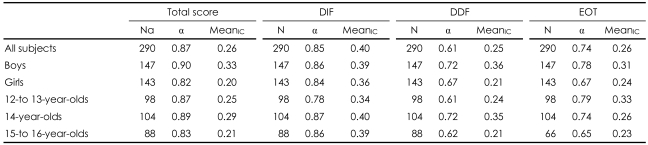
Internal consistency (Cronbach's α) and Mean iter-item correlation coefficient (Mean_IC_) for the TAS-20K and the three factors in the whole sample and in boys and girl and in different age-groups

DIF: difficulty in identifying feelings, DDF: difficulty in describing feelings, EOT: externally-oriented thinking, TAS-20K: the Korean version of the 20-Item Toronto Alexithymia Scale, α: Cronbach's α

**TABLE 4:**
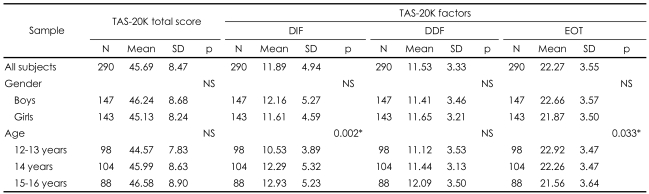
Descriptive statistics of the total TAS-20K score and the three factors of TAS-20K for the whole sample and boys and girls and different age-groups

^*^p<0.05. TAS-20K: the Korean version of Toronto Alexithymia Scale, DIF: difficulty in identifying feelings, DDF: difficulty in describing feelings, EOT: externally-oriented thinking, N: number, SD: standard deviation, NS: not significant
